# Impact of Ayurvedic purification (Shodhana) on *Mucuna pruriens* for primary health care in Parkinson’s disease: phytochemical profile, bioavailability of L-DOPA, and comparative UPLC-MS/MS QTOF–Caco-2 analysis

**DOI:** 10.3389/fmed.2026.1889543

**Published:** 2026-10-01

**Authors:** Sushma Naranappa Salethoor, Shyamasundaran Kulangara, Reshma N. S., Rina Barouch Bentov, Gopi Mohan C., Santhwana S. Nair, Rashee Pantawne, Alok Kumar Tiwari

**Affiliations:** 1 Amrita School of Ayurveda, Amrita Vishwa Vidyapeetham University, Amritapuri, India; 2 Kasturba Medical College Manipal, Manipal, India; 3 Department of Comparative Medicine, Stanford University, Stanford, CA, United States; 4 Amrita Centre for Nanosciences and Molecular Medicine, Amrita Vishwa Vidyapeetham, Kochi, India

**Keywords:** *Mucuna pruriens*, *Kapikacchu*, Parkinson’s disease, L-DOPA, *Godugdha Shodhana*

## Abstract

Parkinson’s disease (PD) is an increasing global health challenge, and although L-DOPA remains the cornerstone of treatment, its long-term use in synthetic form is associated with complications such as dyskinesia and wearing-off. In Ayurveda, *Mucuna pruriens* (*Kapikacchu*/MP) is used in conditions similar to PD. However, the effects of classical purification methods and different dosage forms on their phytochemical composition and functional outcomes have not been well investigated. The present *in vitro* study evaluated the qualitative phytochemical profiling (untargeted) of traditionally Purified *Kapikacchu Choorna* (PKC), Non-Purified *Kapikacchu Choorna* (NKC), and Market sample *Kapikacchu Choorna* (MKC). Additionally, commonly used Ayurvedic dosage forms – *Kapikacchu Choorna* (KC), *Kashaya* (KK), and *Ksheerapaka* (KKP) – were compared for L-DOPA quantification and bioavailability to assess cellular safety and intestinal transport characteristics using the Caco-2 cell line. UPLC-MS/MS QTOF was used to characterise compositional differences among samples and assess the impact of processing methods on therapeutic potential. A total of 486 unique compounds were identified across the three *Kapikacchu Choorna* formulations with NKC (*n* = 102), PKC (*n* = 344), and MKC (*n* = 129). Among these, the phytochemical profiling identified 51 compounds with reported toxicological associations and 20 compounds with reported neuroprotective properties across all samples, with the exclusive presence of the phytoconstituent L-DOPA 3’-glucoside. The L-DOPA content was 0.022% in NKC, 0.087% in PKC and 0.027% in the MKC. Among the KK and KKP samples, KKP demonstrated the most favorable functional performance, exhibiting higher absolute L-DOPA content both before (82.27% vs. 66.67%) and after (29.29% vs. 22.75%) Caco-2 transport. These findings imply that traditional Ayurvedic milk-based purification and dosage form can significantly influence the phytochemical composition, functional L-DOPA delivery, and therapeutic potential of KC, with KKP emerging as the most promising formulation for L-DOPA delivery in the Caco-2 model. From a public health perspective, this is particularly relevant, as KC is already in use either clinically as a standalone therapy or as part of integrative care for PD, making evidence-based guidance on processing methods and dosage forms increasingly important.

## 1 Introduction

Over the past three decades, Parkinson’s disease (PD) has emerged as a major contributor to the global neurological burden and is now recognized as a snowballing neurological disorder in terms of prevalence, disability, and mortality (1). The Global Burden of Disease study estimates show that global PD prevalence increased from 3.15 million cases in 1990 to 11.77 million cases in 2021 (2), with forecasts indicating 25.2 million cases by 2050, a 112% increase from 2021 levels (3). Since the 1960s, since the launch of L-3,4-dihydroxyphenylalanine (levodopa; L-DOPA) in clinical practice, it has remained the cornerstone of the management of PD (4). However, long-term synthetic L-DOPA therapy narrows the therapeutic window and leads to motor complications, including “wearing-off” fluctuations and levodopa-induced dyskinesia (LID) (5, 6). Dyskinesia is a symptom that often occurs after prolonged treatment, with approximately 40–50% of patients developing it within the first 5 years, impairing the quality of life significantly (7).

In Ayurveda, *Kapikacchu – Mucuna pruriens* (MP) seeds have long served for conditions such as PD (8). The findings from studies reveal that it possesses anti-Parkinsonian and neuroprotective effects, which may be attributed, at least in part, to its antioxidant activity (9). MP contains a high concentration of L-DOPA (10) as compared to standard L-DOPA medication used to treat PD (11, 12). In addition to L-DOPA, MP preparations contain L-tyrosine and L-phenylalanine, which are relevant to catecholamine synthesis; theoretically, they may support dopamine production in remaining dopaminergic neurones, though their clinical significance in PD remains uncertain (13).

In MP, along with L-DOPA, the important antinutritional factors include tannins, trypsin inhibitors, saponins, and alkaloids. Antinutritional factors are chemical compounds synthesized in plants that interfere with the absorption of nutrients such as proteins, vitamins, and minerals (14). These compounds may impair digestion and cause adverse effects depending on the plant part consumed and the quantity ingested. Ingestion of crude Mucuna pruriens seeds has been associated with neurological and digestive symptoms (15), including L-DOPA-like adverse effects (16–18) and toxic psychosis (19). A major challenge in toxicology is the accurate identification and evaluation of the causative agent, particularly in cases of plant-based poisoning. Processing of Mucuna pruriens seeds has been reported to reduce several anti-nutritional factors, including tannins, trypsin inhibitor activity, saponins, alkaloids, phytates, phenols and cyanogenic compounds, although the degree of reduction depends on the method used (20).

Processing methods, such as soaking raw seeds for 15 h followed by boiling for 90 min, can reduce L-DOPA content by approximately 70% compared to dried seeds (21). L-DOPA is also reported to degrade during soaking, autoclaving, roasting, sprouting, and alkaline fermentation (22), whereas acidic roasting, ultrasound-assisted extraction with acid and heat, aqueous citric acid extraction, and co-processing with *Phyllanthus emblica* may improve L-DOPA yield (23–26).

Ayurveda highlights that certain medicinal plants require a purification process, termed ‘*Shodhana*’, prior to their safe therapeutic use. For MP, this process, by tradition, involves boiling the seeds in specific media, such as cow’s milk *(Godugdha)* (27). Modern scientific investigations on *Shodhana* procedures applied to various medicinal plants have illustrated that these processes can bring about significant chemical transformations which may influence the pharmacological activity (28). While numerous research studies have examined extraction methods, phytochemical profiling, and the bioavailability of MP, to our knowledge, no studies have evaluated its chemical profile and L-DOPA content following the Ayurvedic method of purification *(Shodhana)*, specifically pertaining to the presence or removal of previously reported anti-nutritional factors.

Moreover, the commonly used Ayurvedic dosage forms in the management of Parkinson’s disease (PD), including *Choorna* (powder), *Kashaya* (herbal decoction), and *Ksheerapaka* (milk decoction), are insufficiently examined with respect to their L-DOPA content and their intestinal bioavailability. To resolve this paucity, the present *in vitro* study was designed to comprehensively evaluate the influence of traditional Ayurvedic purification *(Shodhana)* and dosage form on the phytochemical and functional characteristics of MP.

UPLC–MS/MS QTOF-based phytochemical profiling was employed to characterize the phytochemical composition and quantify L-DOPA in purified, non-purified, and market samples of KC powder (Mucuna pruriens). The intestinal transport and bioavailability of L-DOPA were then investigated using the Caco-2 cell line model, facilitating a comparative evaluation of the two commonly used Ayurvedic dosage forms – Kashaya and Ksheerapaka. The cytotoxicity of the formulations was subsequently assessed through the MTT assay. In addition, computational analyses, including the identification of potential neuroprotective compounds, compound–target interaction mapping, network pharmacology, pathway enrichment analysis of neuroprotective compound targets, and toxicity risk profiling of the three formulations, were performed. Our study also investigated whether purification and processing methods could reduce or eliminate antinutritional and toxic factors while enhancing the consistency, safety, and therapeutic potential of MP formulations, which are used in both standalone Ayurvedic treatment and integrative approaches for PD management.

## 2 Materials and methods

### 2.1 Procurement and purification of the raw materials

A single batch of *Mucuna pruriens* (MP) seeds was procured from a raw-drug store, and the market sample of *Kapikacchu* powder was obtained from a GMP-certified Ayurvedic pharmacy. The seeds were washed; floating seeds were discarded; and the selected seeds were air-dried for 2 days to remove surface moisture, then dried overnight under controlled laboratory conditions. Cow milk was obtained from routine household milking in the hamlet.

Traditional purification (*Shodhana*) as prescribed in Ayurvedic text was carried out using *Dola Yantra Swedana* – a classical steaming/boiling procedure in which the drug is suspended in a cloth bundle (*Potali*) in a liquid medium. The seeds were tied in a cloth bundle and boiled in 450 ml of milk for 3 h, maintaining the temperature between 89 °C and 98 °C. After cooling, the seed coats were removed, and the purified seeds were dried thoroughly, pulverized into fine powder, and stored in airtight containers.

A single batch of three Ayurvedic dosage forms of MP was then prepared following classical procedures. *Choorna* was prepared by pounding and grinding the seeds into fine powder. *Kashaya* was prepared from coarse *Kapikacchu Choorna* using water in a 1:16 ratio and reducing it to one-eighth, as described in the classical formulary text *Sharangadhara Samhita*. *Ksheerapaka* was prepared using the classical 1:8:32 ratio of drug, milk, and water, respectively, and reduced until the milk portion remained, followed by filtration.

### 2.2 MTT assay

#### 2.2.1 Cell culture and MTT assay

L132 and Caco-2 cells (NCCS, Pune) were maintained in Dulbecco’s Modified Eagle’s Medium (DMEM; Sigma) supplemented with 10% fetal bovine serum (FBS; Thermo Fisher, Cat. No. 10270106), 7.5% sodium bicarbonate, and 1% penicillin–streptomycin antibiotic solution (Sigma, India) under standard culture conditions (37 °C, 5% CO_2_, > 90% humidity) in a CO_2_ incubator (Esco Technologies, USA). Confluent cells were harvested using 0.25% trypsin–EDTA (Thermo Fisher, Cat. No. 25200056) and seeded into multi-well plates for subsequent experiments. All procedures were performed under aseptic conditions using a laminar flow hood.

Cell viability was initially assessed by morphological observation using an inverted phase-contrast microscope (Olympus CKX41), followed by quantitative analysis using the MTT (TC191, HiMedia) assay.

#### 2.2.2 Preparation of test samples for cytotoxicity assays

For L132 cell line cytotoxicity testing, KC samples (NKC, PKC, and MKC) were dissolved in autoclaved Milli-Q water to obtain a stock solution of 1 μL/mL. The solution was sterilized by filtration through a 0.22 μm syringe filter (Millipore) and added to the culture medium to achieve final concentrations of 6.25, 12.5, 25, 50, and 100 μg/mL. Culture medium without a test sample served as the control. For the Caco-2 cell line, KC samples were dissolved in 5% DMSO (TC185, HiMedia) to prepare a stock solution. The solution was sterilized by filtration (0.22 μm, Millipore) and added to the culture medium to achieve final concentrations ranging from 6.25 to 100 μL/mL. Culture medium without a test sample served as the control. Three samples were tested in triplicate.

#### 2.2.3 Cytotoxicity assay

Direct microscopic observation method: The entire slab was observed, and images were recorded after 24 h of treatment using an inverted phase-contrast microscope. detectable changes in morphology, including cell rounding or shrinkage, as well as cytoplasmic granulation and vacuolation, were considered as indicators of cytotoxicity.

#### 2.2.4 MTT method

MTT (15 mg) was reconstituted in 3 mL PBS (phosphate-buffered saline) until completely dissolved and sterilized by filtration. The contents of the wells were removed, and 30 μL of reconstituted MTT solution was added to all test and control wells after a 24 h incubation period. The slab was gently shaken and incubated at 37 °C in a humidified 5% CO_2_ incubator for 4 h. The supernatant was removed following incubation, and 100 μL of MTT solubilization solution (dimethyl sulfoxide, DMSO) was added. The wells were gently mixed to solubilize the formazan crystals. A microplate reader at 540 nm was used to measure absorbance.

Cell viability was calculated using the following formula:


%Viability=(MeanODofsamples×100)/

Mean⁢OD⁢of⁢control⁢group

### 2.3 LC–MS/MS QTOF analysis

#### 2.3.1 Qualitative phytochemical analysis and L-DOPA quantification of KC samples using UPLC–MS/MS QTOF

Qualitative phytochemical profiling (untargeted) and L-DOPA quantification of MP samples were performed using single-injection UPLC–MS/MS QTOF analysis across one batch of three formulations: NKC, PKC, and MKC. This comparative analysis facilitated the evaluation of variations in L-DOPA content among non-purified, purified, and market samples, providing insights into the effect of purification on phytochemical composition. Chromatographic separation was accomplished using a C18 column (2.1 × 50 mm, 1.8 μm) maintained at 22°C with a flow rate of 0.3 mL/min. The mobile phase comprised formic acid in water (A) and methanol (B) in an isocratic ratio of 98:2, with a total runtime of 10 min. Mass spectrometric analysis was performed using ESI–MS–QTOF in positive ion mode under optimized conditions, including a gas temperature of 349°C, gas flow rate of 8 L/min, capillary voltage of 5000 V, nebulizer pressure of 40 psi, and a fragmentor voltage of 175 V.

#### 2.3.2 Sample preparation

A 100 mg sample was extracted with 1:1 (0.01 M HCl: Methanol) solution using 30 min ultrasound sonication and subjected to thermomixer for 1 h at 60 degrees at 1000 rpm. The supernatant solution was collected and evaporated to dryness in a vacuum concentrator, and the extract was reconstituted in 1:1 methanol solution. It is then diluted to a sufficient concentration, syringe-filtered (0.22 microns) and analyzed by LCMS QTOF along with L-DOPA CRM. The area of the standard and the percentage of L-DOPA were calculated.


PercentageofL-DOPA=(SampleArea/StandardArea)×

(StandardWeight/StandardDilution)×(SampleDilution/

SampleWeight)×PurityofStandard

#### 2.3.3 L-DOPA bioavailability and quantification from KC dosage forms using Caco-2 cells

L-DOPA bioavailability and cellular uptake were evaluated using LC–MS/MS QTOF analysis across three KC preparations (KC, KKP, and KK). To measure cellular uptake, the L-DOPA concentration of these formulations was quantified prior to *in vitro* screening in Caco-2 cells (specifically KC, KKP, and KK lines). This methodology allowed contrasting L-DOPA bioavailability across formulations and its uptake at the cellular level.

##### 2.3.3.1 Sample preparation

For KC samples, 15 mL of filtered sample was transferred to a separating funnel, mixed with 20 mL petroleum ether (60–80 °C), and shaken to allow phase separation. The aqueous layer was collected and washed twice with petroleum ether to remove lipids. Subsequently, 25 mL acetonitrile was added, and the mixture was filtered and concentrated under vacuum to dryness. The extract was reconstituted in 1:1 methanol solution, diluted to an appropriate concentration, filtered through a 0.22 μm syringe filter, and analyzed by LC–MS/MS QTOF. For KKP and KK samples, 30 mg of sample was dissolved in 1:1 methanol solution and subjected to serial dilution. The final solution was filtered through a 0.22 μm syringe filter and transferred to LC–MS vials for analysis. For KKPand KK samples from Caco-2 experiments, aliquots were diluted in 1:1 methanol solution, filtered (0.22 μm), and analyzed by LC–MS/MS QTOF.

The transport study was performed in the apical-to-basolateral direction, with the test formulations added to the apical compartment and L-DOPA quantified in the basolateral compartment after transport across differentiated Caco-2 monolayers to mimic intestinal absorption. No decarboxylase inhibitor (e.g., carbidopa or benserazide) was included in the present transport experiments. However, it should be noted here that the endogenous aromatic amino acid decarboxylase (AADC) and catechol-O-methyltransferase (COMT) activities in Caco-2 cells may metabolize a proportion of intracellular L-DOPA, potentially leading to an underestimation of the amount of transported intact L-DOPA transport rather than a complete absorption-metabolism balance.

### 2.4 Data interpretation

Compound identification was carried out by matching accurate mass (<5 ppm error), isotope pattern, retention time, and MS/MS fragmentation spectra against databases including mzCloud, HMDB, METLIN, and ChemSpider, with compounds accepted at a ≥70% match score and putative identifications assigned based on accurate mass and database matching. The 486 unique compounds were subsequently classified into chemical classes using compound (29) name keyword analysis and cross-referenced with ClassyFire and ChemOnt ontologies. LC–MS/MS QTOF-identified compounds were subjected to *in silico* toxicity screening. Classification was based on cross-referencing international regulatory databases (IARC, WHO, EU REACH, EPA, Codex, JECFA) and integrating criteria such as regulatory classification, mechanism and severity of toxicity, and strength of toxicological evidence. Pharmacological annotation of the 16 compounds common to all three formulations was performed through systematic literature searches in PubMed, Scopus, and Web of Science, and evidence levels were graded as strong, moderate, or weak based on the quality and quantity of available data. SwissTargetPrediction and PharmMapper software, along with manual curation, were utilized for this. Subsequently, targets were mapped to disease pathways using KEGG, Reactome, and DisGeNET databases, helping in identifying compound–target interactions. The statistical analysis was carried out in Python 3.9 using pandas, matplotlib, and seaborn data libraries. Basic descriptive statistics helped to evaluate compound counts and chemical classes, while a Venn diagram helped to spot which compounds were shared and which were unique.

## 3 Results

### 3.1 Cytotoxicity assessment of NKC and PKC in L132 cell line and KK and KKP in Caco-2 cell line

NKC induced a dose-dependent decline in L132 cell viability (81.34% to 37.83%), with an LC50 of 29.21 μg/mL, indicating significant cytotoxicity. In contrast, purified KC (post-Shodhana) showed higher cell viability (91.72% to 42.13%) with an increased LC50 of 41.93 μg/mL (43.5% rise), reflecting reduced cytotoxicity.

KK showed a gradual, concentration-dependent decrease in cell viability, from 91.42% at 6.25 μg/mL to 47.88% at 100 μg/mL. In contrast, KKP exhibited a more pronounced cytotoxic effect, with viability declining from 87.76% to 39.84% across the same concentration range. The LC50 of KK was 88.92 μg/mL, indicating lower cytotoxicity, whereas KKP showed a significantly lower LC50 of 48.13 μg/mL, reflecting higher cytotoxic potency (refer Figure 1).

**FIGURE 1:**
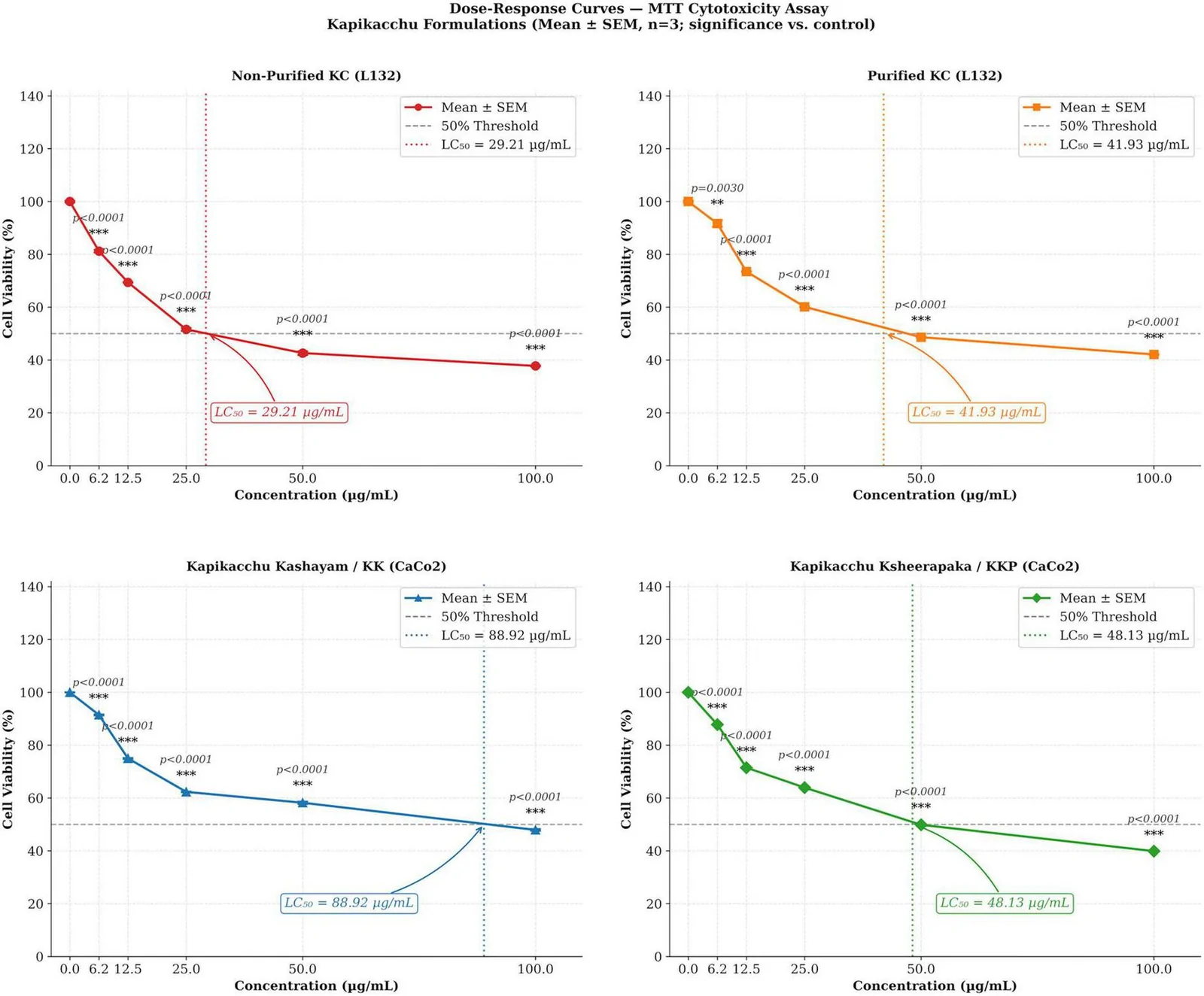
Dose-response curves – MTT cytotoxicity assay Kapikacchu formulations (Mean ± SEM, *n* = 3; significance vs. control).

### 3.2 UPLC MS/MS QTOF analysis

#### 3.2.1 Phytochemical profiling of NKC, PKC and MKC using UPLC-MS/MS QTOF

##### 3.2.1.1 Overall and shared compound counts across formulations

LC-MS/MS QTOF analysis identified a total of 486 unique compounds across the three KC formulations. Among these, PKC showed the highest compound count (*n* = 344), followed by MKC (*n* = 129) and NKC (*n* = 102). The number of compounds detected uniquely within each formulation was highest in PKC (*n* = 302), followed by MKC (*n* = 70) and NKC (*n* = 41), while the corresponding shared compound counts within each formulation were 42, 59, and 61, respectively. Pairwise comparison showed that 14 compounds were shared between NKC and PKC, 31 compounds between NKC and MKC, and 12 compounds between PKC and MKC. In addition, 16 compounds were common to all three formulations. The ranked intersection analysis further showed the relative contribution of each unique and shared compound group to the total compound pool (*n* = 486) (refer Figure 2).

**FIGURE 2:**
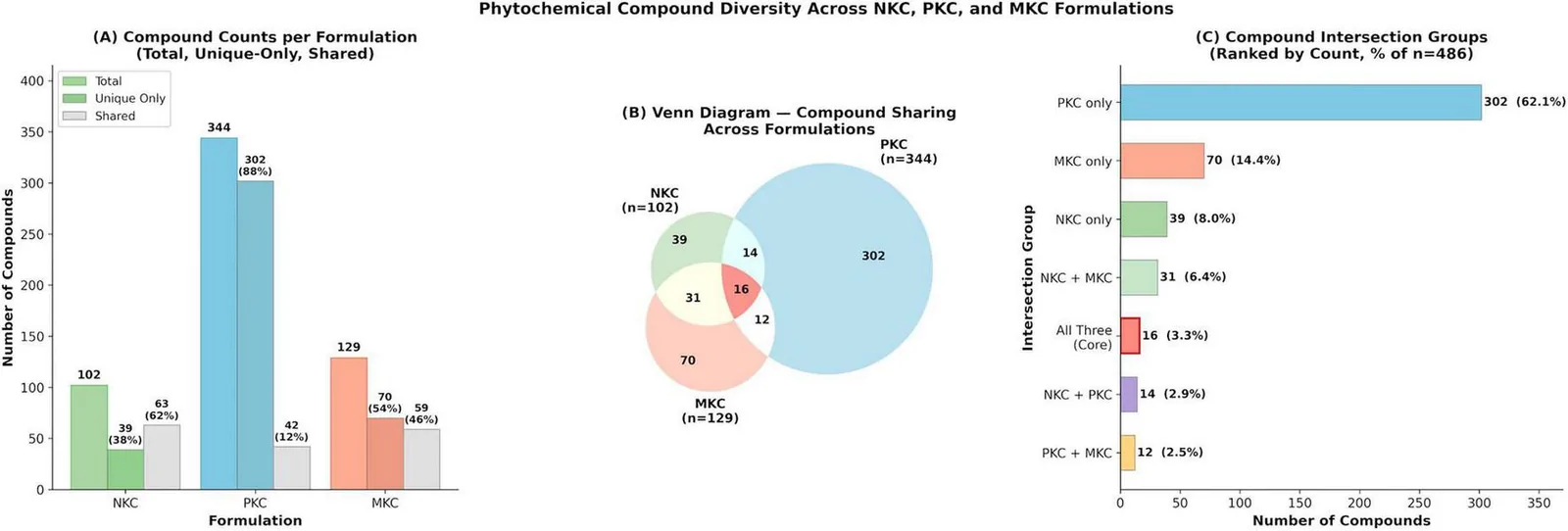
Phytochemical compound diversity across NKC, PKC, and MKC formulations. **(A)** Grouped bar chart showing the total, formulation-specific (unique), and shared compound counts for NKC (*n* = 102), PKC (*n* = 344), and MKC (*n* = 129), with corresponding percentages of the total unique compounds (*n* = 486). **(B)** Three-set Venn diagram illustrating the overlap of phytochemical compounds among the three formulations, with the central intersection (*n* = 16) representing compounds common to all formulations. **(C)** Horizontal bar chart showing the distribution and percentage contribution of each intersection group relative to the total unique compounds (*n* = 486). NKC, non-purified Kapikacchu Choorna; PKC, purified Kapikacchu Choorna (Godugdha Shodhana); MKC, market Kapikacchu Choorna.

##### 3.2.1.2 Functional chemical category classification

To provide a functionally meaningful classification framework beyond conventional chemical class designations, all 486 unique compounds identified across the three KC formulations were assigned to one of nine functional chemical categories (Figure 3). This 8-category system is comprised of Carbohydrates & Sugars, Amino Acids & Derivatives, Fatty Acids & Lipids, Alkaloids & Nitrogenous Compounds, Terpenoids & Polyphenols, Vitamins & Cofactors, Volatile/Flavor & Aroma Compounds, and Industrial/Cytotoxic Compounds.

**FIGURE 3:**
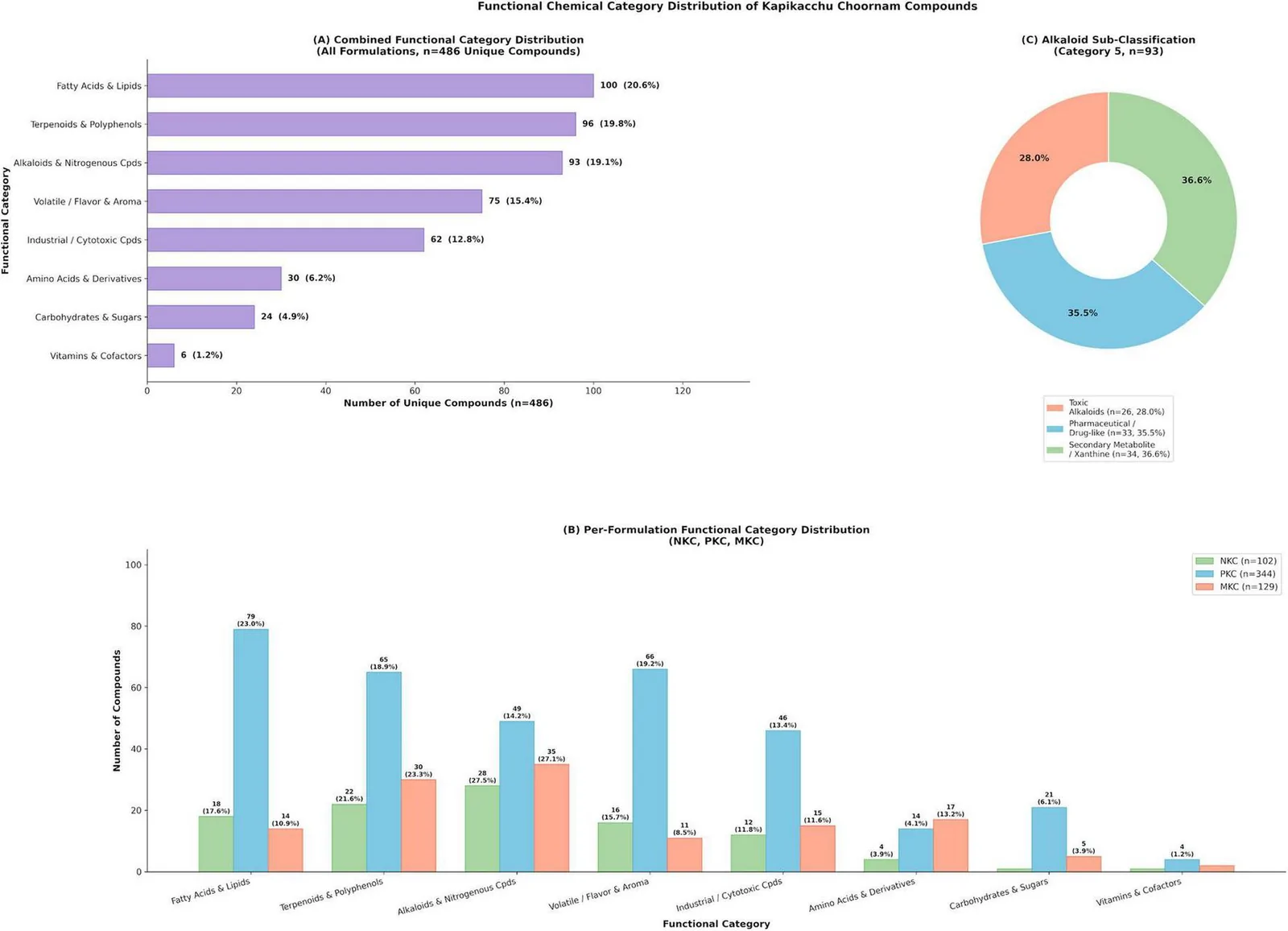
Functional chemical category distribution of Kapikacchu Choorna compounds. **(A)** Horizontal bar chart showing the number of unique compounds (*n* = 486) assigned to each of the eight functional chemical categories across all three KC formulations. **(B)** Grouped bar chart showing the per-formulation distribution of functional chemical categories for NKC (*n* = 102), PKC (*n* = 344), and MKC (*n* = 129). **(C)** Sub-classification breakdown of Category 5 (Alkaloids & Nitrogenous Compounds; n = 93), showing the proportional distribution of Toxic Alkaloids (28.0%), Pharmaceutical/Drug-like Alkaloids (35.5%), and Secondary Metabolite/Xanthine Alkaloids (36.6%).

(A) Horizontal bar chart showing that across the combined dataset of 486 unique compounds, Fatty Acids & Lipids constituted the largest category (*n* = 100, 20.6%), followed by Terpenoids & Polyphenols (*n* = 96, 19.8%), Alkaloids & Nitrogenous Compounds (*n* = 93, 19.1%), and Volatile/Flavor & Aroma Compounds (*n* = 75, 15.4%). Industrial/Cytotoxic Compounds represented (*n* = 62, 12.8%) of the total, while Amino Acids & Derivatives (*n* = 30, 6.2%), Carbohydrates & Sugars (*n* = 24, 4.9%), and Vitamins & Cofactors (*n* = 6, 1.2%) constituted the remaining categories. (B) Stacked bar chart showing the per-formulation distribution of functional chemical categories in which PKC showed the greatest absolute enrichment in Fatty Acids & Lipids (*n* = 79), Volatile/Flavor & Aroma Compounds (*n* = 66), Terpenoids & Polyphenols (*n* = 65), and Industrial/Cytotoxic Compounds (*n* = 46). NKC was proportionally enriched in Alkaloids & Nitrogenous Compounds (28/102, 27.5%). MKC showed the highest proportional representation of Amino Acids & Derivatives (17/129, 13.2%). (C) Sub-classification breakdown of Category 5 (Alkaloids & Nitrogenous Compounds; *n* = 93), showing the proportional distribution of Toxic Alkaloids (28.0%), Pharmaceutical/Drug-like Alkaloids (35.5%), and Secondary Metabolite/Xanthine Alkaloids (36.6%).

##### 3.2.1.3 Milk-derived compound profile across NKC, PKC, and MKC

Comparative analysis of milk-derived versus seed-derived compounds across all three KC formulations revealed a prominent enrichment of milk-derived compounds in PKC following *Godugdha Shodhana* (Figure 4). (A) Absolute compound counts of milk-derived vs. seed-derived compounds per formulation: PKC contained 235 milk-derived compounds compared to 58 in NKC and 66 in MKC. (B) Proportional composition (%) of milk-derived vs. seed-derived compounds – milk-derived compounds accounted for 68.1% of all PKC compounds, compared to 56.9% in NKC and 51.2% in MKC. (C) Per-formulation origin donut charts showing total compound counts. (D) Chemical class analysis of milk-derived compounds revealed that fatty acids and lipids were the dominant class in PKC (*n* = 25), followed by alkaloids (*n* = 5) and glycosides/saponins (*n* = 5), with smaller contributions from terpenoids (*n* = 1) and lignans (*n* = 1). In NKC, fatty acids/lipids (*n* = 3), alkaloids (*n* = 1), and glycosides/saponins (*n* = 3) were the predominant milk-derived classes. MKC showed an intermediate profile with fatty acids/lipids (*n* = 4), alkaloids (*n* = 3), and glycosides/saponins (*n* = 5). (E) Retention time distribution analysis of milk-derived compounds showed that PKC milk-derived compounds eluted predominantly at 32–33 min, while NKC and MKC showed similar but lower-intensity distributions at 33 min. (F) Fold enrichment analysis confirmed that PKC has a 4.05 × enrichment of milk-derived compounds relative to NKC (baseline 1.00×), while MKC showed only a modest 1.14 × enrichment.

**FIGURE 4:**
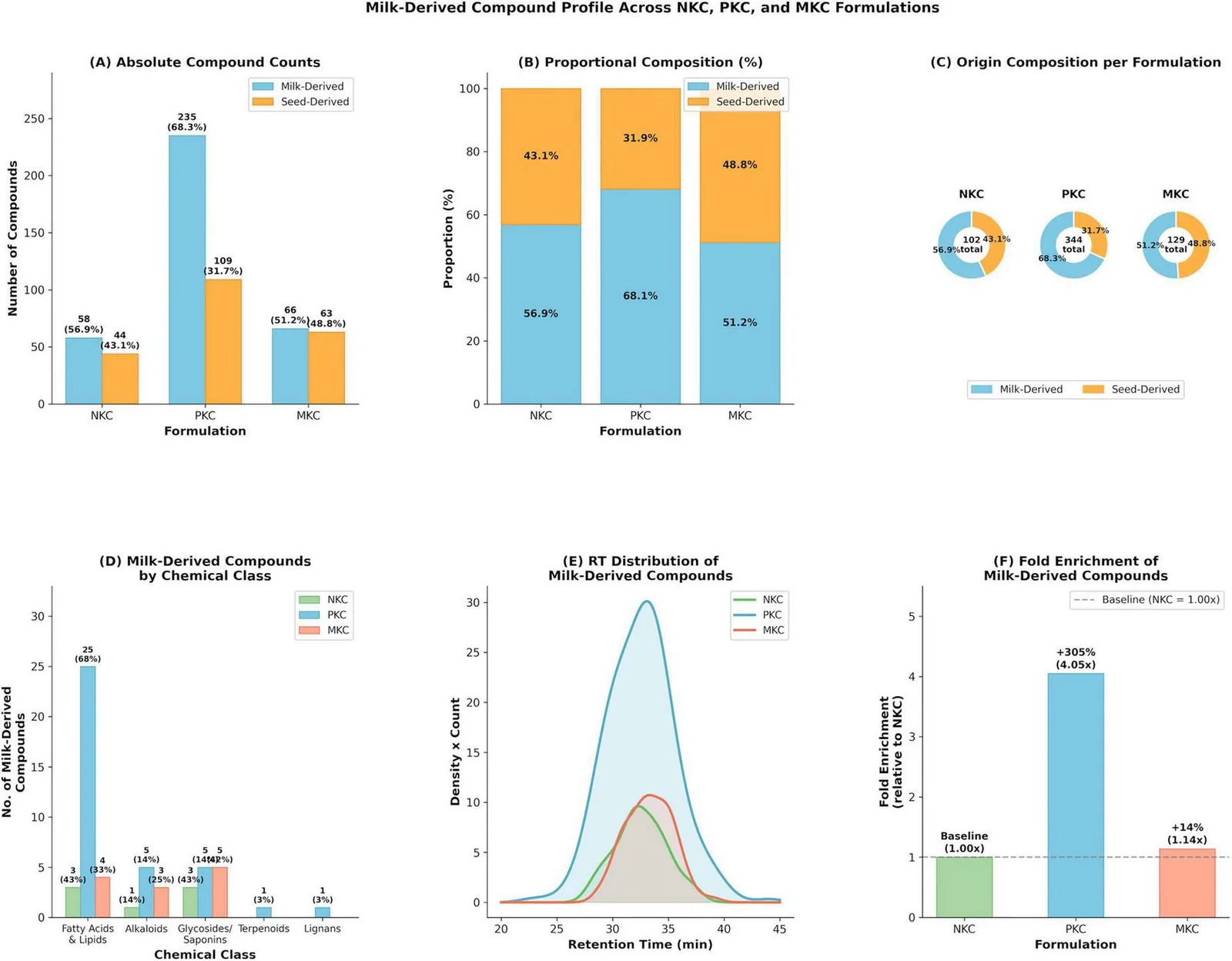
Milk-derived compound profile across NKC, PKC, and MKC formulations. **(A)** Absolute compound counts of milk-derived vs. seed-derived compounds per formulation. **(B)** Proportional composition (%) of milk-derived vs. seed-derived compounds. **(C)** Per-formulation origin donut charts showing total compound counts. **(D)** Milk-derived compounds by chemical class across formulations. **(E)** Retention time distribution of milk-derived compounds (density x count). **(F)** Fold enrichment of milk-derived compounds relative to NKC baseline (1.00x). PKC shows 4.05x enrichment attributable to Godugdha Shodhana purification.

##### 3.2.1.4 Toxicity risk profile across NKC, PKC, and MKC formulations

Comparative *in silico* toxicity risk assessment across all three KC formulations revealed distinct toxicity profiles that reflect both the shared phytochemical core and the formulation-specific compound complements. Across all three formulations, NKC sample contained 102 total compounds, of which 9 were classified as toxic (8.8%). PKC exhibited markedly higher chemical diversity, with 344 compounds including 36 toxic constituents (10.5%). MKC contained 129 compounds, with 21 identified as toxic, representing the highest toxicity proportion (16.3%).

A total of 51 compounds with reported toxicological associations were putatively identified through LC–MS/MS QTOF-based phytochemical profiling of the three formulations. Five compounds were identified as shared across all three formulations, constituting the irreducible toxicological baseline of KC: naphthalene dihydrodiol, cis-1,2-dihydroxy-1,2-dihydro-8-methylnaphthalene, ethyl acrylate, benzothiazole and diisobutyl phthalate. An additional 3 compounds were shared between NKC and MKC (phloroglucinol, cycluron, o-toluidine), 1 compound (diethyltoluamide/DEET) was shared between NKC and PKC, and 1 compound (anhalonidine) was shared between PKC and MKC. NKC contained 9 toxic compounds, including ethyl acrylate, naphthalene dihydrodiol, cis-1,2-dihydroxy-1,2-dihydro-8-methylnaphthalene, o-toluidine, benzothiazole, diisobutyl phthalate, cycluron, diethyltoluamide (DEET), and phloroglucinol. PKC exhibited the highest number of toxic compounds (36 compounds), comprising the compounds common to NKC together with additional metabolites such as dihydroceramide, lauroyl peroxide, T-2 toxin, oleandrin, cucurbitacin B, avermectin A1a, domoic acid, psorospermin, fumitremorgin C, demethoxyfumitremorgin C, N’-nitrosonornicotine, Glu-P-1, MPTP analog, anthanthrene, 2,4-dichloroaniline, formetanate, gamma-coniceine, L-DOPA 3’-glucoside, homocysteinesulfinic acid, skatole, octhilinone, DEHA, di-n-hexyl phthalate, coronatine, solanocapsine, crotamiton, anhalonidine, glucobrassicin, glucocaffeic acid, and methyl caffeate.

MKC contained 21 toxic compounds, including ethyl acrylate, naphthalene dihydrodiol, cis-1,2-dihydroxy-1,2-dihydro-8-methylnaphthalene, dinotefuran, diuron, Yellow AB, o-toluidine, benzothiazole, diisobutyl phthalate, pirimicarb, fenazaquin, cycluron, 3”-hydroxy-geranylhydroquinone, anhalonidine, baptifoline, buxamine E, phloroglucinol, 3-hexylpyridine, imazaquin, saccharin, and threo-syringoylglycerol. The identified compounds represented diverse toxicity categories, including genotoxic, carcinogenic, neurotoxic, cytotoxic, immunotoxic, cardiotoxic, endocrine-disrupting, and pesticide-related compounds. Notably, although PKC contained the highest absolute number of putatively toxic compounds, interpretation should be made cautiously because the present study was based on untargeted LC–MS/MS QTOF phytochemical profiling, and the compound annotations represent putative identifications derived from accurate mass, isotope pattern, fragmentation matching, and database searches rather than confirmation using authentic reference standards (refer to Table 1).

**TABLE 1:** Toxic compounds identified across NKC, PKC, and MKC formulations of *Kapikacchu Choorna* (KC).

#	Compound name	Molecular formula	Formulation(s)	Toxicity category
1	Ethyl acrylate	C5 H8 O2	NKC, PKC, MKC	Genotoxic/Carcinogenic
2	Naphthalene dihydrodiol	C10 H10 O2	NKC, PKC, MKC	Genotoxic/Carcinogenic
3	cis-1,2-Dihydroxy-1,2-dihydro-8-methylnaphthalene	C11 H12 O2	NKC, PKC, MKC	Genotoxic
4	Dihydroceramide	C19 H39 N O3	PKC	Cytotoxic/Pro-apoptotic
5	Lauroyl peroxide	C24 H46 O4	PKC	Oxidative/Cytotoxic
6	Mycotoxin T-2	N/A	PKC	Mycotoxin/Immunotoxic
7	Oleandrin	C32 H48 O9	PKC	Cardiotoxic
8	Cucurbitacin B	C32 H46 O8	PKC	Cytotoxic/Hepatotoxic
9	Avermectin A1a	C49 H74 O14	PKC	Neurotoxic/Veterinary drug residue
10	Domoic acid	C15 H21 N O6	PKC	Neurotoxic/Excitotoxic
11	Psorospermin	C19 H16 O6	PKC	Genotoxic/Cytotoxic
12	Fumitremorgin C	C22 H25 N3 O3	PKC	Mycotoxin/Neurotoxic
13	Demethoxyfumitremorgin C	C21 H23 N3 O2	PKC	Mycotoxin/Neurotoxic
14	N’-Nitrosonornicotine	C9 H11 N3 O	PKC	Carcinogenic/Genotoxic
15	Glu-P-1 (2-Amino-6-methyldipyrido[1,2-a:3’,2’-d]imidazole)	N/A	PKC	Carcinogenic/Genotoxic
16	1-Methyl-4-phenyl-1,2,3,6-tetrahydropyridine (MPTP analogue)	N/A	PKC	Neurotoxic/Parkinsonian
17	Anthanthrene	C22 H12	PKC	Carcinogenic/Genotoxic
18	2,4-Dichloroaniline	C6 H5 Cl2 N	PKC	Carcinogenic/Haematotoxic
19	Formetanate	C11 H15 N3 O2	PKC	Pesticide residue/Neurotoxic
20	Dinotefuran	C7 H14 N4 O3	MKC	Pesticide residue/Neurotoxic
21	Diuron	C9 H10 Cl2 N2 O	MKC	Pesticide residue/Carcinogenic
22	Yellow AB (p-aminoazobenzene)	N/A	MKC	Carcinogenic/Genotoxic
23	o-Toluidine	C7 H9 N	NKC, MKC	Carcinogenic/Genotoxic
24	gamma-Coniceine	C8 H15 N	PKC	Neurotoxic/Teratogenic
25	L-DOPA 3’-glucoside	C15 H21 N O9	PKC	Neurotoxic/Cardiovascular (excess dose)
26	Benzothiazole	C7 H5 N S	NKC, PKC, MKC	Environmental contaminant/Cytotoxic
27	Diisobutyl phthalate	C16 H22 O4	NKC, PKC, MKC	Plasticiser/Endocrine disruptor
28	Homocysteinesulfinic acid	C4 H9 N O4 S	PKC	Neurotoxic/Cardiovascular
29	3-Methylindole (Skatole)	N/A	PKC	Pulmonary toxin/Cytotoxic
30	Octhilinone (OIT)	N/A	PKC	Cytotoxic/Allergenic
31	Di(2-ethylhexyl) adipate (DEHA)	N/A	PKC	Plasticiser/Endocrine disruptor
32	Di-n-hexyl phthalate	C20 H30 O4	PKC	Plasticiser/Endocrine disruptor
33	Coronatine	C18 H25 N O4	PKC	Phytotoxin/Immunomodulatory
34	Solanocapsine	C27 H46 N2 O2	PKC	Neurotoxic/Cytotoxic
35	Crotamiton	C13 H17 N O	PKC	Pharmaceutical contaminant
36	Pirimicarb	C11 H18 N4 O2	MKC	Pesticide residue/Neurotoxic
37	Fenazaquin	C20 H22 N2 O	MKC	Pesticide residue/Mitochondrial toxin
38	Cycluron	C11 H22 N2 O	NKC, MKC	Pesticide residue/Herbicide
39	Diethyltoluamide (DEET)	N/A	NKC, PKC	Neurotoxic/Environmental contaminant
40	3″-Hydroxy-geranylhydroquinone	C16 H22 O3	MKC	Cytotoxic/Pro-oxidant
41	Anhalonidine	C12 H17 N O3	PKC, MKC	Neurotoxic/Psychoactive
42	Baptifoline	C15 H20 N2 O2	MKC	Neurotoxic/Cytotoxic
43	Buxamine E	C26 H44 N2	MKC	Cytotoxic/Neurotoxic
44	Glucobrassicin	C16 H20 N2 O9 S2	PKC	Anti-nutritional/Goitrogenic
45	Phloroglucinol	C6 H6 O3	NKC, MKC	Pro-oxidant/Cytotoxic
46	Glucocaffeic acid	C15 H18 O9	PKC	Pro-oxidant/Genotoxic
47	Methyl caffeate	C10 H10 O4	PKC	Pro-oxidant/Cytotoxic
48	3-Hexylpyridine	C11 H17 N	MKC	Cytotoxic/Environmental contaminant
49	Imazaquin	C17 H17 N3 O3	MKC	Pesticide residue/Herbicide
50	Saccharin	C7 H5 N O3 S	MKC	Artificial sweetener
51	threo-Syringoylglycerol	C11 H16 O6	MKC	Pro-oxidant/Anti-nutritional

Toxic compounds – *Kapikacchu Choorna* (KC) LC-MS/MS QTOF phytochemical profiling.

##### 3.2.1.5 Neuroprotective compounds

LC–MS/MS QTOF-based phytochemical profiling identified a total of 20 unique compounds with reported neuroprotective potential across the three KC formulations.

NKC contains three neuroprotective compounds, namely *paraxanthine*, *valine*, and *cervonyl carnitine*.

PKC exhibited the highest diversity, with 14 neuroprotective compounds, including paraxanthine, valine, inulobiose, 1-methyladenosine, isopentenyl adenosine, isopropyl β-D-glucoside, L-DOPA 3’-glucoside, L-hexanoylcarnitine, 4-aminobenzoic acid (PAB), phytosphingosine, pseudouridine, sapropterin, stearic acid, and stearoylethanolamide.

MKC contained eight neuroprotective compounds, namely *paraxanthine, valine, inulobiose, linoleoyl ethanolamide, sesamol, urocanic acid*,γ*-linolenic acid*, and γ*-tocopherol*.

Among these, paraxanthine and valine were common to all three formulations, whereas inulobiose, a compound associated with the gut–brain axis, was shared between PKC and MKC. Functional categorization revealed that the identified compounds were associated with multiple neuroprotective pathways, including neurodegeneration-related mechanisms (paraxanthine, 1-methyladenosine, isopentenyl adenosine, L-DOPA 3’-glucoside, pseudouridine, and sapropterin), metabolic and lipid regulation (valine, cervonyl carnitine, L-hexanoylcarnitine, phytosphingosine, and stearic acid), gut–brain axis modulation (inulobiose, isopropyl β-D-glucoside, and 4-aminobenzoic acid), and neuroinflammatory pathways (stearoylethanolamide, linoleoyl ethanolamide, sesamol, urocanic acid, γ-linolenic acid, and γ-tocopherol). Overall, PKC demonstrated the greatest diversity of putative neuroprotective metabolites, particularly those linked to neurodegeneration, metabolic regulation, and gut–brain axis interactions. However, as these compounds were identified through untargeted LC–MS/MS QTOF-based metabolite annotation; the findings represent putative identifications and require confirmation using authentic reference standards and targeted analytical approaches (refer to Table 2).

**TABLE 2:** Summary of all 20 neuroprotective compounds identified across KC formulations.

#	Compound name	Neuroprotective pathway category
Shared (All 3) (2 compounds)		
1	Paraxanthine	Neurodegeneration
2	Valine	Metabolic and Lipid
Shared (PKC + MKC) (1 compound)		
3	Inulobiose	Gut-Brain Axis
Unique to NKC (1 compound)		
4	Cervonyl carnitine	Metabolic and Lipid
Unique to PKC (11 compounds)		
5	1-Methyladenosine	Neurodegeneration
6	Isopentenyl adenosine	Neurodegeneration
7	Isopropyl beta-D-glucoside	Gut-Brain Axis
8	L-DOPA 3’-glucoside	Neurodegeneration
9	L-Hexanoylcarnitine	Metabolic and Lipid
10	PAB/4-Aminobenzoic acid	Gut-Brain Axis
11	Phytosphingosine	Metabolic and Lipid
12	Pseudouridine	Neurodegeneration
13	Sapropterin	Neurodegeneration
14	Stearic acid	Metabolic and Lipid
15	Stearoylethanolamide	Neuroinflammation
Unique to MKC (5 compounds)		
16	Linoleoyl Ethanolamide	Gut-Brain Axis
17	Sesamol	Neuroinflammation
18	Urocanic acid	Neuroinflammation
19	γ-Linolenic Acid	Neuroinflammation
20	γ-Tocopherol	Neuroinflammation

Neuroprotective compounds – *Kapikacchu Choorna* (KC) LC-MS/MS QTOF phytochemical profiling. Total: 20 unique neuroprotective compounds | NKC = 3 | PKC = 14 | MKC = 8 | Shared (All 3): Paraxanthine, Valine | Shared (PKC + MKC): Inulobiose.

##### 3.2.1.6 Pathway enrichment analysis of neuroprotective compound targets

Pathway enrichment analysis mapped the 20 identified neuroprotective compounds across four biological domains: Neuroinflammation (5 compounds: stearoylethanolamide, urocanic acid, sesamol, γ-linolenic acid, γ-tocopherol), Neurodegeneration (6 compounds: paraxanthine, L-DOPA 3’-glucoside, sapropterin, pseudouridine, 1-methyladenosine, isopentenyl adenosine), Metabolic & Lipid (5 compounds: cervonyl carnitine, valine, phytosphingosine, stearic acid, L-hexanoylcarnitine), and Gut-Brain Axis (4 compounds: isopropyl beta-D-glucoside, inulobiose, PAB/4-aminobenzoic acid, linoleoyl ethanolamide).

PKC-specific compounds contributed disproportionately to pathway coverage, engaging compounds in all four biological domains (Neurodegeneration: 5 compounds; Metabolic & Lipid: 3 compounds; Gut-Brain Axis: 3 compounds; Neuroinflammation: 1 compound), compared with shared compounds (Neurodegeneration: 1; Metabolic & Lipid: 1; Gut-Brain Axis: 1) and MKC-specific compounds (Neuroinflammation: 4; Gut-Brain Axis: 1). NKC-specific compounds contributed only to the Metabolic & Lipid domain (cervonyl carnitine).

##### 3.2.1.7 Network pharmacology: compound-target interaction map

Network pharmacology analysis mapped 7 key compounds to 11 molecular targets across 4 major disease areas (Figure 5). Paraxanthine showed the richest target profile, interacting with adenosine A1 receptor, adenosine A2A receptor, ryanodine receptor (RyR), and phosphodiesterase (PDE) – all relevant to PD and neurodegeneration. L-DOPA 3’-glucoside (PKC-unique) mapped to dopamine D1/D2 receptors, directly relevant to the dopaminergic deficit in PD. Stearoylethanolamide (PKC-unique) mapped to PPAR-α and endocannabinoid receptors, relevant to neuroinflammation suppression.

**FIGURE 5:**
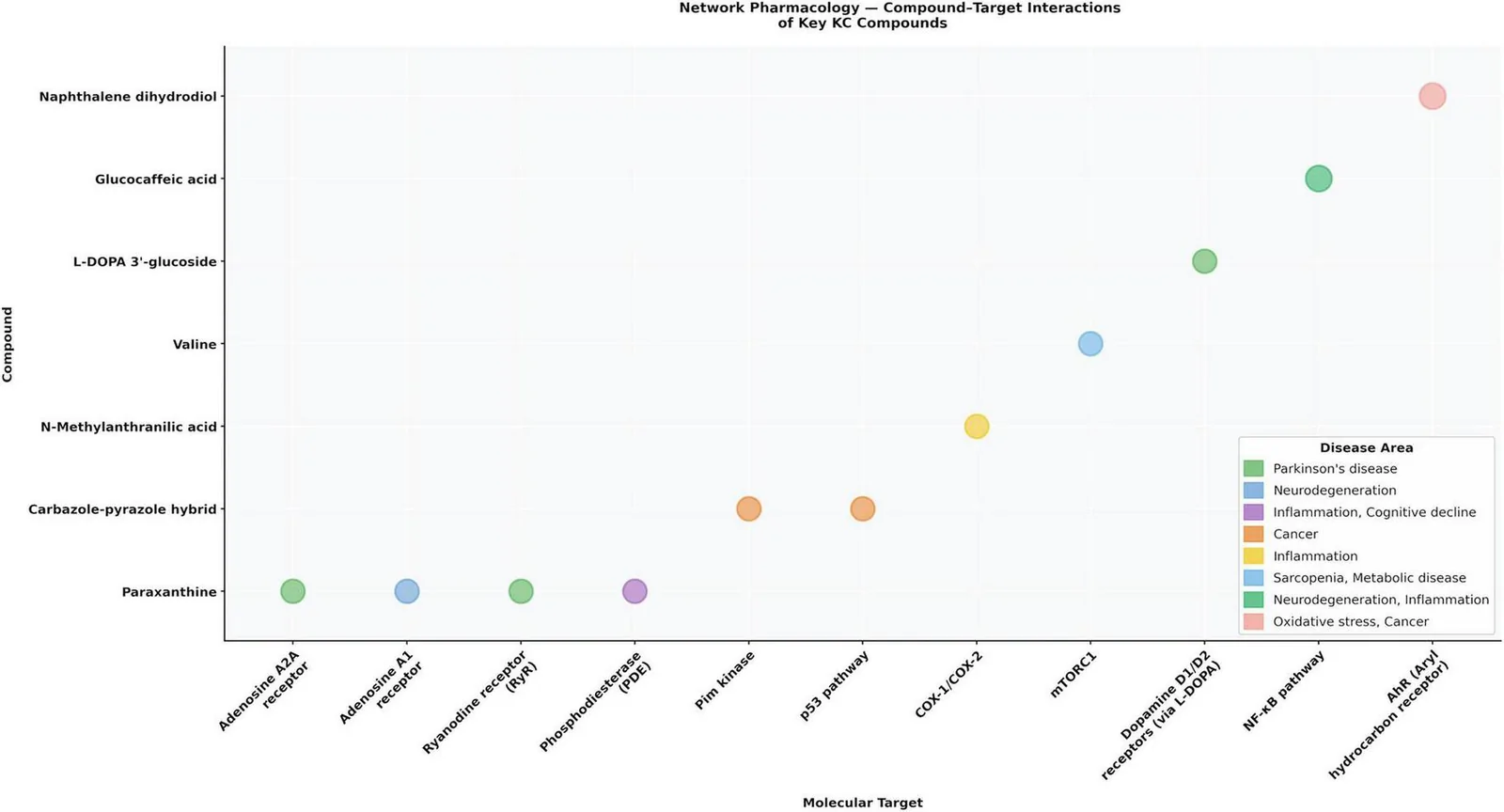
Network pharmacology – compound-target interactions of key KC compounds.

### 3.3 Comparative L-DOPA quantification and bioavailability of KC dosage forms

L-DOPA content increased after *Shodhana*, rising from 0.022% to 0.087% w/w in the *Choorna* preparation, while the market sample showed only a slight increase over the unprocessed sample (0.027% vs. 0.022%). In the decoction-based preparations, *Ksheerapaka* showed a higher L-DOPA content than *Kashaya* (82.27% vs. 66.67%). In the Caco-2 transport model, *Ksheerapaka* and *Kashaya* showed comparable transport efficiency (35.6% and 34.1%, respectively). However, because of its higher initial L-DOPA content, *Ksheerapaka* delivered a greater absolute amount of L-DOPA to the basolateral compartment (29.29% vs. 22.75%). Overall, a 64–66% reduction in L-DOPA was observed during transport (refer Figure 6).

**FIGURE 6:**
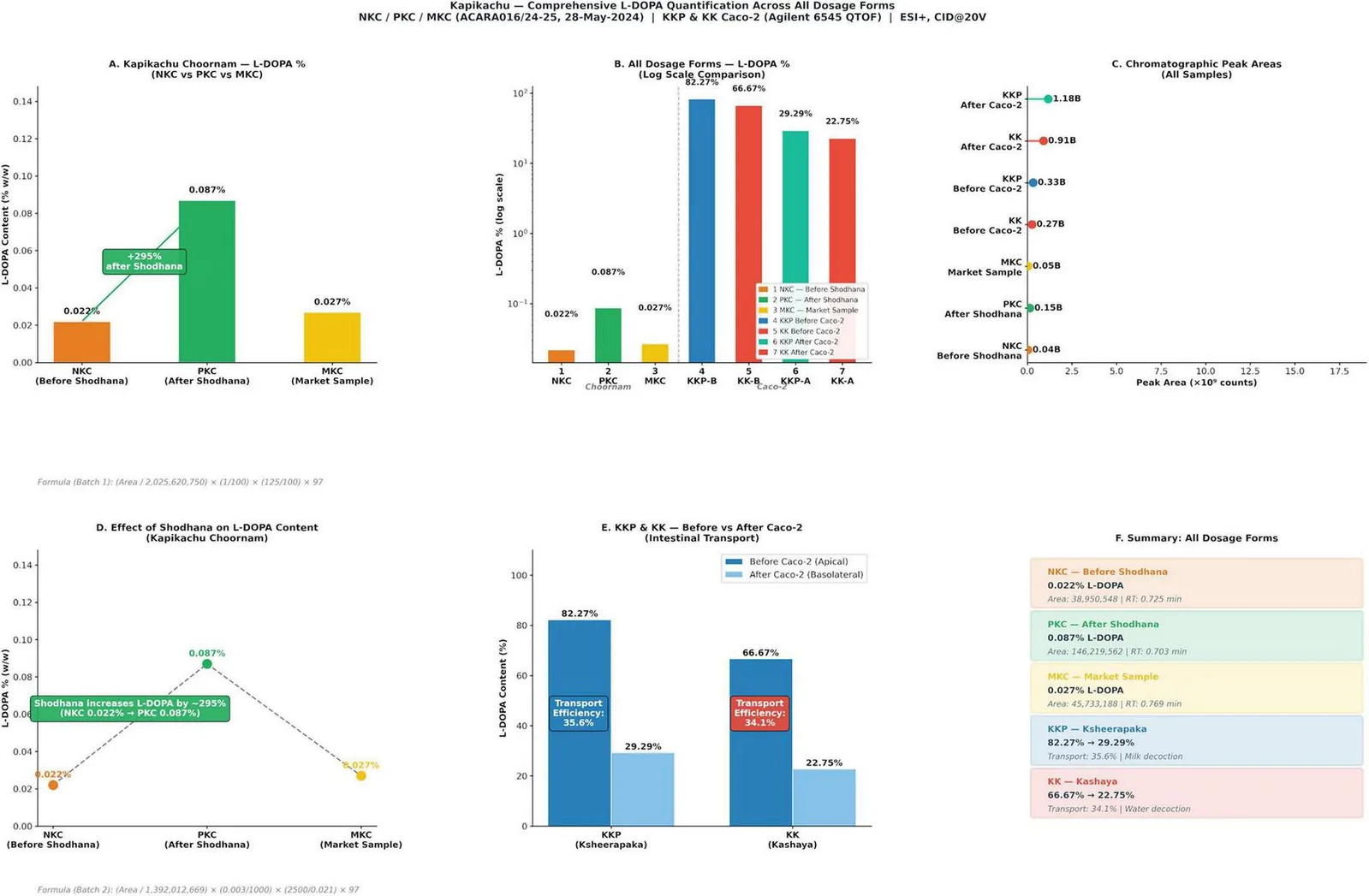
All dosage forms comprehensive comparison. **(A)** Kapikachu Choorna L-DOPA % – before Shodhana, after Shodhana, and market sample with +295% annotation. **(B)** All dosage forms on log scale – spanning Choorna (0.022%–0.087%) to CaCo-2 samples (22.75%–82.27%). **(C)** Chromatographic peak areas for all 7 samples. **(D)** Shodhana effect slope chart – before → after Shodhana → market sample. **(E)** KKP and KK before vs. after CaCo-2 transport with efficiency annotations. **(F)** Summary cards for all dosage forms with key values.

## 4 Discussion

### 4.1 Phytochemical profiling of NKC, PKC and MKC using UPLC-MS/MS QTOF

The present metabolomic analysis provides a comparative phytochemical profile of the three formulations based on putative compound annotations derived from high-resolution mass spectrometry and database matching. Although this approach enables broad characterization of phytochemical differences among formulations, confirmation of selected key compounds using authenticated reference standards and complementary analytical techniques would further strengthen the structural assignments and biological interpretation of the findings. Therefore, these findings should be interpreted within the context of qualitative phytochemical profiling.

After identifying the compounds through UPLC–MS/MS QTOF analysis, we compared the phytochemical profiles of NKC, PKC, and MKC to understand how processing and dosage form may influence their composition. The following sections discuss these findings, focusing on milk-derived compound enrichment, possible toxicity-related signals, neuroprotective compounds, network pharmacology-based mechanisms, and differences in L-DOPA content across the formulations.

#### 4.1.1 Milk-derived compound enrichment across formulations

The higher concentration of milk-derived compounds in PKC compared to NKC corroborates how *Godugdha* (cow’s milk) biochemically alters and influences the *Shodhana* (27, 28). LC–MS/MS QTOF data showed that 235 distinct milk-derived compounds were infused into PKC due to the milk processing, compared to only 58 in NKC and 66 in MKC. The predominance of fatty acids and lipids among milk-derived compounds in PKC (*n* = 25) is consistent with the known lipid-rich composition of bovine milk, which contains approximately 3.5–4.5% fat comprising triglycerides, phospholipids, sphingolipids, and free fatty acids (30, 31). A pharmacologically significant finding was the 5.5-fold enrichment of fatty acids and lipids in PKC (109 compounds, 31.7%) compared with NKC (20 compounds, 19.6%) and MKC (19 compounds, 14.7%). Fatty acids and lipids are increasingly recognized as bioactive constituents with neuroprotective, anti-inflammatory, and membrane-stabilizing properties (32–35). Fatty acids such as oleic acid, palmitic acid, and stearic acid – the dominant species in bovine milk fat – modulate neuroinflammatory signaling through PPAR-γ activation and competitive inhibition of arachidonic acid-derived pro-inflammatory eicosanoids (36, 37). The observed synergy in bioavailability is consistent with the Ayurvedic concept of ‘*Samskara*’ (processing-induced transformation of a substance’s properties), as described in the classical Ayurvedic text *Charaka Samhita* – *“Samskaro hi gunantaradhanam”*, thereby modifying its pharmacological attributes, including absorption and efficacy (38). The modest 1.14-fold enrichment of milk-derived compounds in MKC compared to NKC is a critical finding with regard to the quality and direct implications for the standardization of commercial KC formulations. This near-baseline enrichment suggests that MKC has not undergone equivalent *Godugdha Shodhana* processing, resulting in a substantially reduced milk-derived phytochemical complement compared to classically prepared PKC. The present data provide quantitative LC-MS/MS evidence that such simplifications result in a measurably inferior phytochemical profile, particularly with respect to milk-derived lipids, alkaloids, and glycosides that contribute to the neuroprotective and bioavailability-enhancing properties of classically prepared KC. These findings support the classical *Godugdha Shodhana* as an essential quality-determining step in KC preparation and highlight the need for standardized processing protocols for commercial Ayurvedic formulations (39, 40). Based on the experimental results, using computational predictions, the toxicity endpoint flags were observed in MKC compared to PKC – particularly for cytotoxicity (21.7% vs. 14.2%) and neurotoxicity (14.7% vs. 8.7%). The neurotoxicity flag rate in MKC is particularly concerning given that KC is primarily used for neurological indications (41), and neurotoxic contaminants could potentially counteract the intended neuroprotective effects (42, 43). These findings provide a rationale for preferring classically prepared, *Godugdha Shodhana*-purified KC over commercial formulations for therapeutic use and support the implementation of standardized toxicological screening as part of quality control for commercial Ayurvedic KC products.

Seventeen PKC-specific compounds introduced were identified following *Godugdha Shodhana* – including oxidized lipids, Maillard reaction products, ceramide derivatives, sterol oxides, and the unexpected hemlock alkaloid gamma-coniceine (44–46). Oxidized lipids and sterol oxides, while potentially pro-inflammatory at high concentrations, are ubiquitous in thermally processed foods and are generally considered safe at dietary exposure levels (33). The detection of gamma-coniceine the most acutely toxic hemlock alkaloid, a nicotinic acetylcholine receptor antagonist causing neuromuscular blockade and respiratory paralysis – is the most significant toxicological finding in PKC and warrants further identification confirmation before clinical interpretation (44–46). Its presence in an MP-derived formulation is biologically implausible and most likely reflects either raw material contamination with Conium maculatum (poison hemlock) or a false-positive identification at the LC-MS/MS matching threshold; both scenarios require further investigation. With the exception of gamma-coniceine, the remaining PKC-specific compounds represent a manageable and largely well-characterized toxicological profile that does not undermine the overall safety advantage of Godugdha Shodhana-purified KC. (Supplementary file)

#### 4.1.2 Toxicity risk profile across NKC, PKC, and MKC formulations

The comparative toxicity analysis provides critical safety insights into the three KC formulations. Phytochemical profiling across all samples identified a total of 51 toxic compounds (47–103). Detailed compound-level data are provided in the Supplementary File.

Based on computational analysis, we could identify that the NKC sample comprised 102 total compounds, with 9 identified as toxic constituents (8.8%). The PKC sample exhibited a more expansive chemical profile of 344 compounds, which contained 36 identified as toxic species (10.5%). The MKC sample consisted of 129 compounds, 21 of which were categorized as toxic, yielding the highest toxicity proportion (16.3%). This finding gives clear evidence of how *Godugdha Shodhana* (purification using cow’s milk) steps up to improve safety. While classical Ayurvedic texts have long described *Shodhana* as a way to detoxify by transforming or removing harmful compounds in Ayurvedic medicines (27, 28), this LC–MS/MS-based analysis provides an empirical quantitative validation.

Five compounds were identified as shared across all three formulations (NKC, PKC, MKC), constituting the irreducible toxicological baseline of KC: naphthalene dihydrodiol (47, 48) (genotoxic/carcinogenic PAH metabolite), ethyl acrylate (49) (genotoxic/carcinogenic industrial chemical; IARC Group 2B), cis-1,2-dihydroxy-1,2-dihydro-8-methylnaphthalene (50) (genotoxic PAH metabolite), benzothiazole (51) (environmental contaminant), and diisobutyl phthalate (52) (plasticizer/endocrine disruptor).

Subsequent verification using analytical approaches and authentic standards would be valuable to confirm the identity and toxicological impact of these compounds within KC formulations. Detailed compound-level data is provided in the Supplementary File.

#### 4.1.3 Neuroprotective compound profile and multi-mechanism neuroprotection

The current research landscape in PD increasingly emphasizes the exploration of plant-derived neuroprotective compounds as potential therapeutic agents. Supporting this trend, saffron (*Crocus sativus* L.) metabolites such as crocetin, crocins, and safranal have been identified as promising neuroprotective molecules, demonstrating antioxidant, anti-inflammatory, and anti-α-synuclein aggregation activities. Such observations highlight the potential of phytochemicals to target multiple pathogenic pathways in PD and reinforce the rationale for investigating traditional medicinal plants as sources of novel neuroprotective interventions. Within this broader context, MP occupies a unique position owing to its natural L-DOPA content, diverse bioactive phytoconstituents, and long-standing use in traditional systems of medicine for conditions resembling Parkinsonism, making it a particularly relevant candidate for further neuroprotective investigation (53).

Based on the computational analysis, we could identify 20 neuroprotective compounds (104–121) (mentioned in the Supplementary file) across the three KC formulations, representing a comprehensive phytochemical characterization of the neuroprotective potential of MP to date. Previous studies have focused predominantly on L-DOPA as the primary neuroprotective constituent of MP (41, 122, 123), with limited attention to the broader phytochemical matrix. Also, to our knowledge, there have been no previous studies that have examined the Ayurvedic purification method.

The present study demonstrates that KC contains a diverse array of neuroprotective compounds spanning dopaminergic, anti-neuroinflammatory, antioxidant, metabolic, mitochondrial, and gut-brain axis mechanisms, suggesting potential molecular mechanisms underlying the multi-target neuroprotective activity attributed to this formulation in Ayurvedic medicine. The two compounds shared across all three formulations – paraxanthine and valine – constitute the invariant neuroprotective core of KC shared across all three formulations. Paraxanthine is a well-characterized adenosine A1/A2A receptor antagonist that provides dopaminergic neuroprotection through ryanodine receptor (RyR2) activation and cAMP/cGMP signaling (104). Valine, a branched-chain amino acid present in all three formulations, activates mTORC1/S6K1 signaling to support neuronal protein synthesis, synaptic plasticity, and muscle protein anabolism – consistent with the traditional use of KC as a *Rasayana* (rejuvenating tonic) (105).

The eleven PKC-specific neuroprotective compounds represent a clinically significant finding of this study, as they are uniquely introduced by *Godugdha Shodhana*. L-DOPA 3’-glucoside is a glycosylated prodrug form of L-DOPA that may provide more sustained and controlled dopaminergic supplementation compared to free L-DOPA, potentially reducing peak-dose dyskinesia – a major limitation of conventional L-DOPA therapy (111). Sapropterin (BH4), the essential cofactor for tyrosine hydroxylase, directly supports dopamine biosynthesis and is clinically used in dopamine-responsive conditions (116). L-Hexanoylcarnitine supports mitochondrial fatty acid β-oxidation via CPT1/CPT2 transport, maintaining neuronal energy homeostasis in conditions of metabolic stress (112). Stearoylethanolamide, an endocannabinoid-like N-acylethanolamine, reduces neuroinflammation via PPAR-α activation and modulates gut-brain axis signaling (37). Phytosphingosine, a sphingolipid precursor, regulates ceramide-dependent autophagy and mitochondrial apoptosis pathways increasingly recognized as therapeutic targets in neurodegeneration (114). Stearic acid activates PPAR-γ and competitively inhibits arachidonic acid-derived pro-inflammatory eicosanoids, providing anti-neuroinflammatory activity (36). Pseudouridine (115), 1-methyladenosine (108), and isopentenyl adenosine (109) are modified nucleosides that modulate adenosine receptor signaling and RNA stability under oxidative stress conditions. PAB/4-aminobenzoic acid serves as an antioxidant and cofactor for folate synthesis, supporting neuronal one-carbon metabolism (113). Isopropyl beta-D-glucoside may enhance the oral bioavailability of co-administered phytochemicals through glucosidase-mediated hydrolysis (110). Collectively, these PKC-specific compounds – spanning dopaminergic, mitochondrial, sphingolipid, endocannabinoid, and adenosinergic mechanisms – provide a compelling molecular rationale for the enhanced neuroprotective efficacy of *Godugdha Shodhana*-purified KC compared to the non-purified formulation.

The five MKC-specific neuroprotective compounds – γ-tocopherol, γ-linolenic acid, linoleoyl ethanolamide, urocanic acid, and sesamol – suggest that the commercial formulation retains meaningful neuroprotective activity through a distinct mechanistic profile. γ-Tocopherol provides superior protection against peroxynitrite-mediated nitrosative stress compared to α-tocopherol, which is relevant to the oxidative pathology of Parkinson’s disease (121). Urocanic acid, a histidine metabolite, modulates neuroimmune responses through histamine receptor signaling (119). Sesamol, a phenolic lignan, exerts antioxidant and anti-neuroinflammatory effects through NF-κB inhibition and Nrf2 activation (118). However, the absence of the PKC-specific neuroprotective compounds – particularly L-DOPA 3’-glucoside, sapropterin, stearoylethanolamide, and the gut-brain axis modulators (isopropyl beta-D-glucoside, PAB/4-aminobenzoic acid) – in MKC suggests that commercial formulations that have not undergone proper Godugdha Shodhana lack the full neuroprotective phytochemical complement of classically prepared KC. These findings have direct implications for the quality standardization of commercial Ayurvedic KC formulations and support the scientific validation of classical *Shodhana* procedures as essential for maximizing the therapeutic potential of MP-based medicines (27, 39).

#### 4.1.4 Network pharmacology and multi-target mechanisms

Based on network pharmacology analysis, we identified 11 compound-target pairs across 7 compounds and 4 major disease pathways, revealing the multi-target pharmacological profile of KC. This multi-target profile is consistent with the Ayurvedic concept of *Rasayana* – a formulation that promotes overall health through multiple complementary mechanisms rather than a single molecular target. The convergence of multiple KC compounds on PD-relevant targets [adenosine A1/A2A receptors (104), dopamine D1/D2 receptors (111), RyR, PDE (104)] provides a compelling molecular rationale for the traditional use of KC in *Kampavata* (Ayurvedic comparison for Parkinson’s disease). Correlating valine with mTORC1 activation supports the mechanistic basis for using KC as a nutritional tonic in managing sarcopenia and muscle wasting. Branched-chain amino acids, including valine, are well-known activators of mTORC1-mediated protein synthesis (105), their detection in KC provides a plausible explanation for its use in Ayurvedic practice as a tissue-building and strengthening agent.

### 4.2 Quantification of L-DOPA in different MP dosage forms based on experimental analysis

#### 4.2.1 L-DOPA content in ksheerapaka and kashaya dosage forms

It was found that *Ksheerapaka* (KKP) outperformed *Kashaya* (KK) significantly, yielding 82.27% L-DOPA compared to KK’s 66.67% – a striking 23.4% increase. The enhancement could likely be driven by the presence of proteins, lipids, and casein micelles, which can bind with L-DOPA, boosting its stability and solubility during preparation. From a clinical standpoint, this higher L-DOPA yield in *Ksheerapaka* is specifically favorable for treating neurological conditions (124), and the results suggest that the milk matrix not only preserves more active compounds but might also deliver its own neuroprotective benefits through its rich protein and calcium profile (125). L-DOPA administered alongside milk proteins may mitigate competitive inhibition against dietary amino acids for intestinal LAT1/LAT2 transporter binding, which could potentially augment net L-DOPA uptake. The absence of an AADC inhibitor in the transport experiments may have permitted intracellular metabolism of L-DOPA within Caco-2 cells. Consequently, the measured concentrations of intact L-DOPA may underestimate the total amount of transported L-DOPA and its metabolites. Although these findings are consistent with active intracellular metabolism of L-DOPA within the Caco-2 model, further targeted studies are needed to directly confirm the specific metabolic pathways involved.

#### 4.2.2 Caco-2 intestinal transport and bioavailability

To predict how well these formulations are absorbed in the gut, a Caco-2 cell monolayer model, which is a well-established *in vitro* system for testing drug absorption (126) was used. KKP and KK performed almost identically during testing, tracking at 35.6% and 34.1%, respectively, showing a minimal 1.5 percentage point variance. These benchmarks mirror the documented clinical findings of oral L-DOPA bioavailability from MP preparations (122). This similarity between KKP and KK indicates that intestinal L-DOPA absorption is largely unaffected by the extraction medium itself. Instead, both formulations appear to actively utilize the same LAT1/LAT2 amino acid transporter pathways to achieve intestinal uptake.

However, despite tracking with similar transport efficiencies, KKP managed to deliver 28.7% more absolute L-DOPA to the basolateral (systemic) compartment (29.29% vs. 22.75%). This variance is likely driven by the higher baseline L-DOPA loading in KKP (82.27% vs. 66.67%). From a clinical application standpoint, this indicates that KKP can deliver 29% more bioavailable L-DOPA than KK, offering a meaningful way for optimizing dopaminergic therapy.

It was noted that L-DOPA levels dropped by 64.4% for KKP and 65.9% for KK during Caco-2 transport, indicating simultaneous cellular uptake and metabolism. This is also driven specifically via aromatic amino acid decarboxylase (AADC/DOPA decarboxylase) and O-methylation via catechol-O-methyltransferase (COMT) (127). Our metabolomics analysis of KC *Choorna* inside the cells confirmed this active internal processing and glucosylation, as we detected L-DOPA 3’-glucoside (score 98.09, RT 1.594–2.425 min). This conversion aligns with standard intestinal Phase II conjugation reactions in intestinal epithelial cells. Therapeutically, these outcomes confirm that KKP manages to supply a larger pool of bioavailable L-DOPA than KK.

Although KKP demonstrated greater absolute basolateral L-DOPA delivery compared with KK, this finding should be interpreted together with the MTT results, which showed a lower LC50 for KKP and therefore higher apparent cytotoxicity in the Caco-2 assay. Thus, KKP may be considered promising specifically for L-DOPA delivery within the experimental Caco-2 model; however, its effects on Caco-2 cell viability warrant cautious interpretation and further studies aimed at dose optimization and safety evaluation before any therapeutic conclusions can be drawn.

**Strength of the study**

A key strength of this research lies in its unified approach to evaluating MP by seamlessly bridging phytochemical analysis, functional *in vitro* assessment, and pharmacological interpretation. Furthermore, the study goes beyond exclusively measuring L-DOPA levels. By investigating how *Godugdha Shodhana* affects the plant and directly comparing standard preparations like *Choorna, Kashaya*, and *Ksheerapaka*, it delivers insights that practitioners can apply in real-world Ayurveda/integrative clinical settings.

While UPLC-MS/MS QTOF provided in-depth profiling of post-processing chemical changes, the MTT assay and Caco-2 models provided vital, functional data on safety and how these compounds move through the intestines. Rather than relying solely on chemical composition, evaluating NKC, PKC, and MKC allowed a direct comparison of both classical processing methods and clinically popular formulations.

Additionally, bringing in network pharmacology helped in connecting the identified compounds directly to biological targets connected to PD, neurodegeneration, and inflammation. This provides mechanistic clarity to traditional assertions. On the whole, this study bridges the gap between Ayurvedic pharmaceutical principles and modern analytical and biological tools, creating data that is scientifically robust while remaining deeply rooted in tradition.

**Limitations**

The present investigation was designed as an untargeted qualitative phytochemical profiling study and did not include quantitative or semi-quantitative assessment of individual metabolites. Determination of relative abundance would require a dedicated metabolomic workflow with appropriate experimental design, including optimized extraction procedures, suitable solvent systems, internal standards, and comparative statistical analysis of peak intensities or peak areas. Therefore, the current findings should be interpreted as qualitative phytochemical profiling only, rather than quantitative metabolomic assessment.

Compound identification was based primarily on mass spectrometry data with a match threshold of at least 70%, and most compounds were not fully confirmed using reference standards or Nuclear Magnetic Resonance (NMR). The analysis was conducted on a single batch, so batch-to-batch variability could not be assessed. The pharmacological relevance and toxicity profiles inferred for the detected compounds were derived from published literature and *in silico* tools and therefore remain predictive rather than experimentally validated. Similarly, the network pharmacology findings are hypothesis-generating and have not yet been confirmed through *in vivo* animal studies or clinical investigations. The detection of ethyl acrylate also requires cautious interpretation, as its source remains uncertain and may relate to processing, packaging, or analytical artifacts. In the transport experiments, the Caco-2 model provided only an *in vitro* estimate and does not fully replicate the complexity of human gastrointestinal physiology or true *in vivo* bioavailability. The transport experiments did not include a decarboxylase inhibitor, such as carbidopa or benserazide, nor were major L-DOPA metabolites quantified. Therefore, the present study provides an estimate of intact L-DOPA transport rather than a complete absorption-metabolism balance of L-DOPA and its metabolites. The market comparison was also limited to a single commercial sample and, therefore, may not reflect the full variability present across marketed formulations. Finally, metabolite profiling was not performed for the *Choorna* preparation, so possible L-DOPA conjugates or related forms may have been missed, which could have led to underestimation of total bioavailable L-DOPA.

**Future directions**

Future research should use focused and semi-quantitative metabolomic analysis using authentic reference standards and supplementary analytical techniques to validate important neuroprotective and toxicologically relevant chemicals discovered in the present work. Specifically, targeted profiling of L-DOPA and its main metabolites, in combination with enzyme inhibition strategies, would provide a more comprehensive view of the intestinal absorption, metabolism and overall disposition of L-DOPA in the Caco-2 model, while further clarifying the contribution of specific phytoconstituents to its bioavailability and biological activity.

Future studies employing *in vivo* pharmacokinetic models and well-designed clinical investigations are warranted to validate these findings and establish the safety, dosing, and clinical relevance of the observed effects.

## 5 Conclusion

This research offers analytical and mechanistic evidence that *Godugdha Shodhana* significantly modifies the phytochemical and functional makeup of MP. Beyond solely enhancing the L-DOPA yields in *Kapikacchu Choorna*, this purification step drives chemical complexity by introducing milk-derived lipids, glycosides, and peptides. Furthermore, it improves the overall extraction of the seed-associated bioactive components. Furthermore, the purified formulation yielded a wider variety of neuroprotective constituents, including the exclusive presence of L-DOPA 3’-glucoside, a derivative offering enhanced pharmacokinetics. Network pharmacology confirmed the therapeutic relevance of these modifications by linking multiple compounds to targets involved in PD, neurodegeneration, and inflammatory pathways. Within the shared compounds identified, paraxanthine stood out as a critical neuroactive constituent owing to its known antagonistic activity against adenosine A2A receptors. Among the tested dosage forms, *Ksheerapaka* (KKP) topped by delivering a higher absolute quantity of L-DOPA across the Caco-2 model. However, *Ksheerapaka* and *Kashaya* exhibited comparable intestinal transport efficiencies, indicating that absorption relies mostly on carrier-mediated mechanisms rather than formulation-specific transport traits. Therefore, the greater basolateral delivery achieved by KKP primarily stems from its superior initial L-DOPA loading.

KKP should be considered promising specifically in terms of L-DOPA delivery within the experimental Caco-2 model. At the same time, its lower LC50 and greater reduction in Caco-2 cell viability indicate the need for cautious interpretation, dose optimization, and further safety evaluation. This statement requires correlation clinically before further claims.

The analysis also revealed a major quality deficit in the commercial market sample, which contained significantly less L-DOPA than the classically processed preparation. This deficit underscores the urgent need for strict and standardized quality control, clear pharmacopeial benchmarks, and maintaining manufacturing consistency across *Kapikacchu* formulations. Broadly, this study establishes a strong scientific rationale for the traditional Ayurvedic preference for *Godugdha Shodhana* processing, specifically in the form of *Ksheerapaka*.

It confirms that classical processing actively enhances therapeutic value rather than merely acting as a purification step. Future research should validate these findings through *in vivo* pharmacokinetic models and controlled clinical trials, accounting for dietary amino acid competition and co-administration with decarboxylase inhibitors. From a public health standpoint, these findings are highly relevant, as MP is frequently utilized by patients with PD either independently or alongside conventional therapies. Because processing techniques and formulation forms alter the absolute amount of L-DOPA available for absorption, identifying optimal preparation and consumption methods is vital. Although this study does not establish definitive clinical parameters for dosing or therapeutic equivalence, it represents a significant step advancement by delivering analytical and mechanistic evidence that favors traditional formulations, particularly *Ksheerapaka*. These conclusions underscore the broader necessity for evidence-based usage, ongoing validation, and clearer guidance to assist patients, physicians, and Ayurvedic practitioners in managing long-term PD care.

## Data Availability

The raw data supporting the conclusions of this article will be made available by the authors, without undue reservation.
